# Selective single-molecule analytical detection of C-reactive protein in saliva with an organic transistor

**DOI:** 10.1007/s00216-019-01778-2

**Published:** 2019-03-28

**Authors:** Eleonora Macchia, Kyriaki Manoli, Brigitte Holzer, Cinzia Di Franco, Rosaria A. Picca, Nicola Cioffi, Gaetano Scamarcio, Gerardo Palazzo, Luisa Torsi

**Affiliations:** 1 Dipartimento di Chimica, Università degli Studi di Bari “Aldo Moro”, 70125 Bari, Italy; 2 CNR, Istituto di Fotonica e Nanotecnologie, Sede di Bari, 70125 Bari, Italy; 3 Dipartimento InterAteneo di Fisica “M. Merlin”, Università degli Studi di Bari “Aldo Moro”, 70125 Bari, Italy; 4 CSGI (Centre for Colloid and Surface Science), 70125 Bari, Italy; 5 The Faculty of Science and Engineering, Åbo Akademi University, 20500 Turku, Finland

**Keywords:** Electrolyte-gated organic thin-film transistor, Single molecule, Wide-field transistors, Analytical sensors, CRP detection, Saliva

## Abstract

In the last decade, saliva has been suggested as non-invasive diagnostic fluid, suitable for clinical use alternatively to blood serum and plasma. However, the clinical applicability of saliva has been hampered so far by the inadequate sensitivity of current methods to detect the lower salivary concentrations of many biomarkers monitored in blood products. Herein, a label-free biosensor based on electrolyte-gated organic thin-film transistor (EGOTFT) has been developed for the detection at the physical limit of C-reactive protein (CRP) in human saliva. CRP is a key relevant biomarker for inflammatory processes and is routinely monitored for many clinical purposes. Herein, an electrolyte-gated thin-film transistor (EGOTFT) has been proposed as a transducer of the biorecognition event taking place at the gate electrode, functionalized with a self-assembled monolayer (SAM) of highly densely packed capturing anti-CRP proteins. Thanks to the SAM, the biosensing platform herein proposed is endowed with ultra-high sensitivity, along with an extremely high selectivity, assessed by measuring the dose curves of CRP interacting with a bovine serum albumin-functionalized gate. Moreover, the biosensing platform is compatible with low-cost fabrication techniques and applicable to the ultra-sensitive detection of a plethora of clinically relevant biomarkers. Therefore, the EGOTFT device herein proposed, being able to operate in physiologically relevant fluids such as saliva, will set the ground to a major revolution in biosensing applications for early clinical detection.

## Introduction

The analysis of human biofluids, such as the blood, urine, saliva, tears, and sweat, yields useful clinical information for the evaluation and monitoring of health and disease states [1]. Lab-on-a-chip systems able to quantify clinically relevant biomarkers in physiological fluids and compatible with point-of-care testing have shown terrific advancements in the last decade, foreseeing a revolution in early clinical diagnostics [2–6]. In this perspective, accurate, compact, easy to use, and possibly non-invasive diagnostics represents the ideal candidate for point-of-care testing, especially for geriatric and pediatric patients and for mass screening, where minimally invasive collection of diagnostic material is a pivotal aspect [7].

Blood products, i.e., the serum and plasma, have been so far the most widespread and conventional biofluids for clinical testing. In fact, blood flows and diffuses through all tissues and organs, collecting by-product of many pathological states. Therefore, the concentrations of specific relevant biomarkers in plasma or serum have been associated with ongoing disease states, leading to a plethora of fundamental clinical applications [8]. However, in the last decade, saliva has been promoted as a non-invasive alternative to blood as diagnostic fluid. Saliva represents a very complex matrix, containing both secretion from salivary glands and non-salivary components, such as metabolites and signal molecules accompanying remote processes [9]. In fact most metabolites, cytokines, proteins, and hormones move by passive filtration into saliva from bloodstream. It has been recently proven that their levels in saliva reflect their blood concentrations [10]. During the last few years, great efforts have been made worldwide to characterize and compare the protein compositions of salivary and blood fluids [11]. Proteomic analyses of saliva have highlighted the presence of over 2000 proteins, approximately 25–30% of which are shared with blood [7]. The significant correspondence in protein content between saliva and plasma suggested that saliva could be suitable as a diagnostic alternative to blood tests. Indeed, the use of saliva as an alternative to blood-based assay offers many advantages. In particular, saliva is readily available from most individuals, can be easily collected, stored, and processed. Moreover, the collection procedure is non-invasive, painless, and cost-effective [12]. However, the diffusion of saliva has clinically relevant biofluid has been hindered so far by the insufficient sensitivity of current analytical methods to detect the lower salivary concentrations of many biomarkers compared to blood products [13]. Therefore, salivary diagnostics applicability requires ultra-highly sensitive biosensing platform, being able to selectively detect a specific biomarker in a complex matrix at physiological concentrations. To the best of our knowledge, the present lack of practical detection methodologies represents a major limiting factor, because most current assays do not provide the necessary sensitivity for the detection of biomarkers at the low, but still patho-physiologically relevant, concentrations in saliva [14, 15]. Among the plethora of biomarkers whose quantification in saliva could potentially contribute to monitor the onset of many pathological states, C-reactive protein (CRP) has attracted a great deal of attention. In fact, CRP has been known since the 1930s as an acute phase protein of the immunoresponse [16]. CRP is a non-glycosylated protein with a molecular weight of c.a. 115 kDa, constituted by five non-covalently bound, identical, and spherical subunits, each one of about 20–28 kDa [17]. These subunits are held together in a circular structure called pentraxin [18]. The CRP blood level is presently routinely checked because of its relevance as biomarker for systemic inflammation arising from immune system stimulation, such as infection and tissue damage [19]. In fact, it is well assessed that blood serum concentrations of CRP rise up to 1000-fold upon acute inflammatory stimulus [14]. Besides, the association between prognosis and CRP levels in patients with cancer is currently well established. In particular, CRP is extensively reported in oncology as a reliable biomarker for survival, cancer risks, and tumor recurrence, impacting on many critical decisions in treatment [20]. Moreover, recent clinical and epidemiological studies have demonstrated the association between inflammation and cardiovascular diseases [21]. Furthermore, it has been demonstrated that CRP serum concentrations in patients with rheumatic disease significantly decrease over the course of successful anti-TNFα therapy [22, 23]. Normal blood serum CRP concentrations range from 9 nanomolar (10^−9^ M, nM) to 40 nM [20]. Since the protein is expressed by hepatocytes only upon stimulus, CRP levels may also be significantly below 9 nM. CRP levels of less than 9 nM, in the range between 9 and 40 nM, and greater than 40 nM have been correlated respectively with low, moderate, and high cardiovascular risk for patients in primary prevention programs [20]. A number of high sensitivity assay to accurately measure CRP down to picomolar level (10^−12^ M, pM) in blood serum has been proposed so far [24–26]. Furthermore, by implementing a sandwich assay through the introduction of aptamer-modified quantum dots, detection of CRP in human serum at a LOD of 43 attomolar (10^−18^ M, aM) has been recently demonstrated [27]. However, to the best of our knowledge, saliva is not yet accessible for an accurate CRP quantification and no method has been presently reported to measure CRP in saliva specimens. In fact, because of its non-lipophilic structure and high molecular weight, CRP shows limited transfer from the blood to saliva [28]. All these call for the development of a label-free ultra-highly sensitive clinical assays [29], capable of tracking a biomarker down to the physical limit and compatible with point-of-care testing. In this perspective, the main barrier is the development of a biosensing platform being able to detect single molecules with a sufficiently large signal to noise ratio [30]. Our group has recently demonstrated the first proof-of-concept of label-free single-molecule detection of immunoglobulins with a millimeter-sized transistor (SiMoT) sensor, based on an electrolyte-gated organic thin-film transistor (EG-OTFT) device [31]. The figures of merits of most relevant analytical methods for CRP detection have been summarized in Table 1. In this article, the CRP single-molecule label-free detection accomplished by means of a millimeter-wide bioelectronic electrolyte-gated transistor is presented. In particular, evidences of the ultimate sensitivity and the high selectivity of the SiMoT approach towards CRP both in phosphate-buffered solution (PBS) and in human saliva, accomplished with a bioelectronic transistor whose gate is biofunctionalized with the anti-CRP capturing antibodies, are herein presented. The SiMoT biosensing platform has been engaged in the detection of CRP in a diluted sample of real human saliva, proving that single-molecule detection is possible also real samples.

**Table 1:** Figures of merits of the most relevant analytical methods for CRP detection

	CRP limit of detection	Selectivity	Assay of real biofluid	Label	Time to result
ELISA [16]	2 nM	High	Blood serum	Label needing	Long
EIS [25]	9 pM	High	No	Label free	Short (few hours)
Paper-based ELISA [26]	13 pM	High	Blood serum	Label needing	Long
Nanoparticle amplified SPR [27]	43 aM	High	Blood serum	Label needing	Inherently long
SiMOT	210 zM (13 ± 4 molecules in 100 μl)	High	Saliva	Label free	Short (few hours)

## Materials and methods

### Materials

Poly(3-hexylthiophene-2,5-diyl), P3HT, regioregularity > 99%, organic semiconductor with molecular weight of about 17.5 kDa (g/mol), has been purchased from Sigma-Aldrich. The organic semiconductor has been used with no further purification. 3-Mercaptopropionic acid (3-MPA), 11-mercaptoundecanoic acid (11-MUA), 1-ethyl-3-(3-dimethylaminopropyl)-carbodiimide (EDC), and N-hydroxysulfosuccinimide sodium salt (sulfo-NHS) were purchased from Sigma-Aldrich and used with no further purification. The anti-C-reactive protein (anti-CRP) produced in mouse is monoclonal antibody (molecular weight *~* 24 kDa) and was purchased from Sigma-Aldrich. Human C-reactive protein (CRP) (molecular weight *~* 115 kDa), from Sigma-Aldrich, is isolated from human fluids (ascitic/pleural). Bovine serum albumin (BSA) has a molecular weight of 66 kDa and was purchased from Sigma-Aldrich and readily used. Water (HPLC grade, Sigma-Aldrich) and ethanol grade puriss. p.a. assay, ≥ 99.8%, were used with no further purification. PBS (Sigma-Aldrich) presents osmolality and ion concentrations matching those of the human body (isotonic). One tablet of PBS has been dissolved in 200 ml of water (HPLC grade) that yields 0.01 M phosphate buffer, 0.0027 M potassium chloride, and 0.137 M sodium chloride, pH 7.4, at 25 °C.

### EGOTFT fabrication

The EGOTFTs have been fabricated starting from a highly n-doped silicon substrate, covered by thermally grown SiO_2_. The SiO_2_ surface was cleaned through a procedure involving sonication in solvents of increasing polarity before proceeding with the electrodes patterning. Source (S) and drain (D) interdigitated electrodes were photo-lithographically defined on the Si/SiO_2_ substrate. Electron-beam evaporated gold (thickness 50 nm) has been then deposited on an adhesion layer of titanium (thickness 5 nm). The distance between two differently biased fingers is the channel length (L), being equal to 5 μm, while the perimeter of each set of equipotential fingers of 1280 μm is the channel width (W). Before the deposition of the organic semiconductor (OSC), the substrate with the interdigitated electrodes was cleaned through a procedure involving sonication in 2-propanol.The transistor channel area covered by the organic semiconductor (OSC) was 6.4 10^−3^ cm^2^. Specifically, a P3HT solution (2.6 mg/ml in chlorobenzene) filtered with 0.2 μm filter was spin coated at 2 × 10^3^ r.p.m. for 20 s and annealed at 90 °C for 15 min. A polydimethylsiloxane well was glued across the interdigitated channels area and was filled with 300 μl of water (HPLC grade) acting as gating medium. A Kapton® foil (area of *~* 0.6 cm^2^) covered by e-beam evaporated gold (50 nm) on titanium (5 nm) served as the gate (G) electrodes. The gate was stably positioned on the water on top of the well in correspondence of the electrodes interdigitated area.

### Gate biofunctionalization protocol

The gate electrodes were cleaned in an ultrasonic bath of 2-propanol for 10 min, rinsed with HPLC water, dried with N_2_, and then treated for 10 min in ozone cleaner. The chemical self-assembled monolayer (chem-SAM) functionalization protocol on the gold surface involving a 10-mM solution of 10:1 M ratio of 3-mercaptopropionic acid (3-MPA) to 11-mercaptoundecanoic acid (11-MUA) has been prepared in ethanol. The cleaned gold surface was immersed in the 3-MPA and 11-MUA solution and kept in the dark under constant N_2_ flux for 18 h at 22 °C [32]. The resulting monolayer will be addressed in the following as the chem-SAM. Afterwards, the carboxylic groups were activated in a 200-mM EDC and 50-mM sulfo-NHS aqueous solution for 2 h at 25 °C. The anti-CRP antibody has been used as biorecognition element. Terminal amine groups on the antibody enable covalent binding to the carboxylic functions of the SAM activated with EDC/sulfo-NHS. Specifically, the gate has been immersed in an anti-CRP PBS solution for 2 h at 25 °C. The solution was composed of 4.2 μM (0.1 mg/ml) of anti-CRP and PBS at a pH of 7.4 and an ionic-strength (*i*_s_) of 162 mM. The layer of anti-CRP attached to the chem-SAM forms the bio-SAM. Afterwards, the unreacted sulfo-NHS groups were subsequently saturated with ethanolamine 1 M in PBS 10 mM for 1 h at 25 °C. Finally, the biofunctionalized gate was immersed in a 1.5 μM (0.1 mg/ml) BSA solution in PBS 10 mM for 1 h at 25 °C. The negative control experiment has been performed by means of a gate electrode functionalized with BSA instead of anti-CRP. After each step of the functionalization protocol, the gate was rinsed thoroughly with the corresponding solvent to remove possible unbounded residues. In the following, the layer comprising both the chem-SAM and the bio-SAM is addressed as SAM.

### Sensing measurements

Sensing measurements have been performed according to the experimental protocol reported elsewhere [37]. The transistor transfer characteristics were measured with a semiconductor parameter analyzer in air and at room temperature (RT, 20–22 °C). Before the sensing measurements have been performed, *I*_D_ has been stabilized by recording subsequent repeated measurements of the transfer curve of the P3HT EGOTFT with a bare gold gate, until three current traces perfectly overlay. A functionalized gate was then mounted on the EGOTFT, instead of the bare gold gate previously used, and incubated (at RT and in the dark) for 10 min in 100 μl of PBS. The gate has been removed from the PBS solution and washed thoroughly with HPLC water, and the *I*_0_*baseline* was registered. Afterwards, the same gate was immersed and incubated for 10 min in 100 μl of the PBS standard solutions of the ligands (CRP) with nominal concentrations ranging from 6 to 6 10^14^ zM. After each incubation in the PBS standard solutions of CRP starting from the more diluted one, the gate was washed thoroughly with PBS and water (HPLC grade) to remove unreacted CRP and the relevant I–V transfer curves were measured, being the *signal*. All the data points reported are averaged over three replicates taken on three different devices with three different gates. The resulting reproducibility error is computed as the relative standard deviation. Three BSA-functionalized gates were employed to measure the CRP negative control dose curves as well.

### Detection in saliva

Detection in saliva samples has been performed according to the experimental protocol reported elsewhere [21]. Saliva samples were collected by passive drool method from a healthy human female volunteer, and written informed consent was received. The collected saliva sample was divided into aliquots of 500 μl and frozen at − 20 °C immediately after collection. Each saliva aliquot was brought to room temperature immediately before use and centrifuged at 1.5 10^3^×*g* for 15 min. The supernatant was taken and progressively diluted 1:10^15^ times in PBS. For each assay, a single anti-CRP functionalized gate was incubated for 10 min in each diluted saliva solution, starting from the more diluted to more concentrated one, following the same protocol used for the sensing measurements performed in PBS standard solutions of CRP. As negative control experiment, a gate functionalized with the BSA instead of anti-CRP was incubated in saliva solutions diluted in PBS.

## Results and discussion

**Fig. 1:**
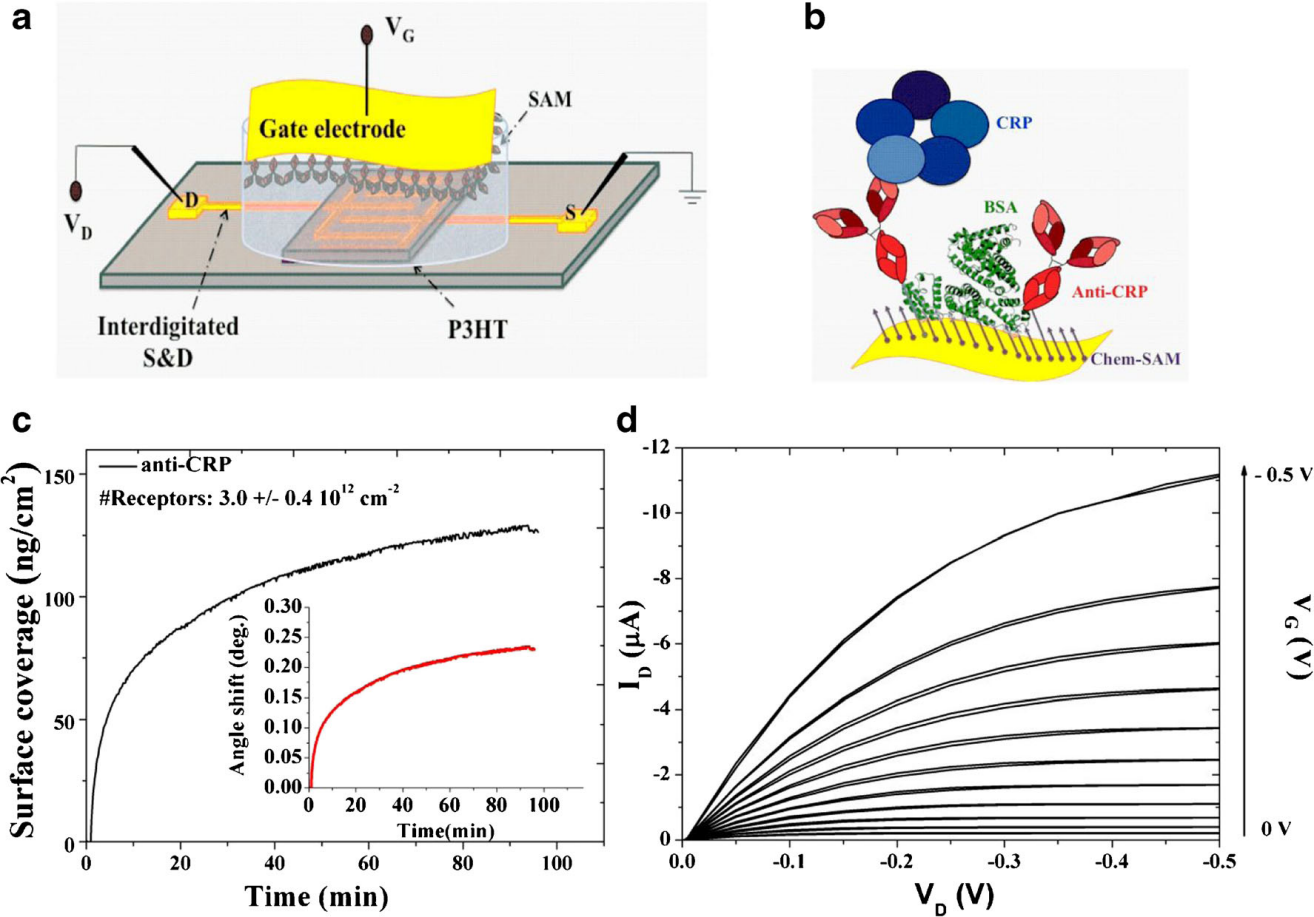
**a** Three-dimensional schematic representation of the developed SiMoT biosensing platform. **b** Sketch of the capturing SAM, representing both the chem-SAM and the bio-SAM of capturing proteins. **c** Anti-CRP surface loading vs. time. Inset: corresponding angular response of the binding of the anti-CRP proteins as a function of time. **d** Typical output curves (*I*_D_ vs. *V*_D_, with *V*_G_ ranging from 0 to − 0.5 V in steps of − 0.1 V) of the SiMoT device

A schematic representation of the biosensing platform based on SiMoT technology herein proposed is reported in Fig. 1a. The SiMoT used in this study is based on an EGOTFT embedding P3HT as organic semiconductor, which forms a conductive channel between source and drain contacts, and water as electrolyte medium. The biorecognition elements, namely anti-CRP, have been immobilized on the gold gate surfaces via self-assembled monolayer (SAM) approach. The formation of covalent bonds between SAMs and the biological recognition sites has been exploited, considering the long-term stability of amide bonds, that show half-lives of roughly 600 years in neutral solution at 25 °C [33]. All details of the biofunctionalization protocols have been already reported elsewhere [30, 34]. Herein, few salient details are recalled. The grafting of chem-SAMs on gold electrode has been achieved through the chemisorption of a mixture of 3-MPA and 11-MUA. A mixture of SAMs with different chain length has been already demonstrated to be necessary for a more effective immobilization of large biomolecules, since biomolecules are able to attach to the surface without undergoing conformational changes due to steric hindrance. As a consequence, thanks to this strategy, an enhanced surface coverage can be obtained [31]. Terminal amine groups on the antibody enable covalent binding to the carboxylic functions of the chem-SAM activated with EDC/sulfo-NHS. Anti-CRP is then immobilized as biorecognition element and subsequently the non-reacted activated acidic functionalities were blocked applying ethanolamine. BSA is finally immobilized to prevent any aspecific binding. The resulting SAM is sketched in Fig. 1b. It has been already demonstrated elsewhere that the amide groups generated with the activation process produce hydrogen bondings between the oxygen of the amide group of one chain to the hydrogen of the amide group of closest neighbor chain [30]. A dipole moment is associated to each H-bond and therefore an electrostatic network is generated. It has been hypothesized that this network of hydrogen bonds is capable of sustaining electrostatic cooperative interaction, likely at the basis of the SiMoT detection at the physical limit.

Surface plasmon resonance (SPR) measurements were first performed to assess the anti-CRP surface coverage of the gold gate electrode. Au-coated (~ 50 nm) SPR slides with a chromium adhesion layer (~ 2 nm) were functionalized with the chem-SAM (3-MPA and 11-MUA, 10:1) prior to SPR measurements according to the protocol used for the gate functionalization. All the steps to subsequently deposit the bio-SAM were accomplished in the SPR cell, holding a volume of 100 μl, by static injection at 22 °C of 300 μl of the relevant solutions.

The amount of anti-CRP, immobilized per unit area can be estimated from the thickness and the refractive index of a non-homogeneous protein layer based on the Feijter equation:

1 $$ \varGamma =\frac{\left({n}_{\mathrm{a}}-{n}_{\mathrm{m}}\right){d}_{\mathrm{a}}}{\frac{\mathrm{dn}}{\mathrm{dc}}} $$

where *n*_a_ is the average refractive index of the antibody layer, *n*_m_ is the refractive index of the buffer solution, *d*_a_ is the average layer thickness and dn/dc is the specific refractivity of the adsorbed antibody layer. Deriving this further to include instrument signal it returns:

2 $$ \left({n}_a-{n}_m\right)=\Delta \theta \times k $$

where *k* is a coefficient for sensitivity depending from the wavelength, while Δ*θ* is the angular response, namely the angle shift in the measurement. Therefore, Eq. 1 becomes:

3 $$ \varGamma =\frac{\Delta  \theta \times k\times {d}_{\mathrm{a}}}{\frac{\mathrm{dn}}{\mathrm{dc}}} $$

For thin layers (< 100 nm) the *k* × *d*_a_ can be assumed constant, being equal to 1.0 × 10^−7^ cm/degree for the laser wavelength in use (*λ* = 670 nm). In accordance with the literature, the dn/dc value for proteins is 0.182 cm^3^/g at 670 nm [35]. Herein, the protein surface coverage is calculated based on the following equation:

4 $$ \varGamma =\Delta  \theta \times 550\ \mathrm{ng}/{\mathrm{cm}}^2 $$

When a 0.1 mg/ml anti-CRP solution in PBS is injected, an angular shift is recorded, as reported in the inset in Fig. 1c. By means of Eq. 4, the surface coverage (*Γ*) can be evaluated from the measured angular shift. The resulting *Γ* values are reported in Fig. 1c and at the equilibrium, the measured anti-CRP coverage is 118.8 ± 14.1 ng/cm^2^. Taking into account the weight of a single anti-CRP protein, the estimated average total number of active binding sites available is 3.0 ± 0.4 10^12^ cm^−2^. Such an extremely large number of capturing proteins packed at the level of 10^5^ · μm^−2^ attached to gate surface make the binding of CRP extremely likely even when the ligands to be detected are very few.

**Fig. 2:**
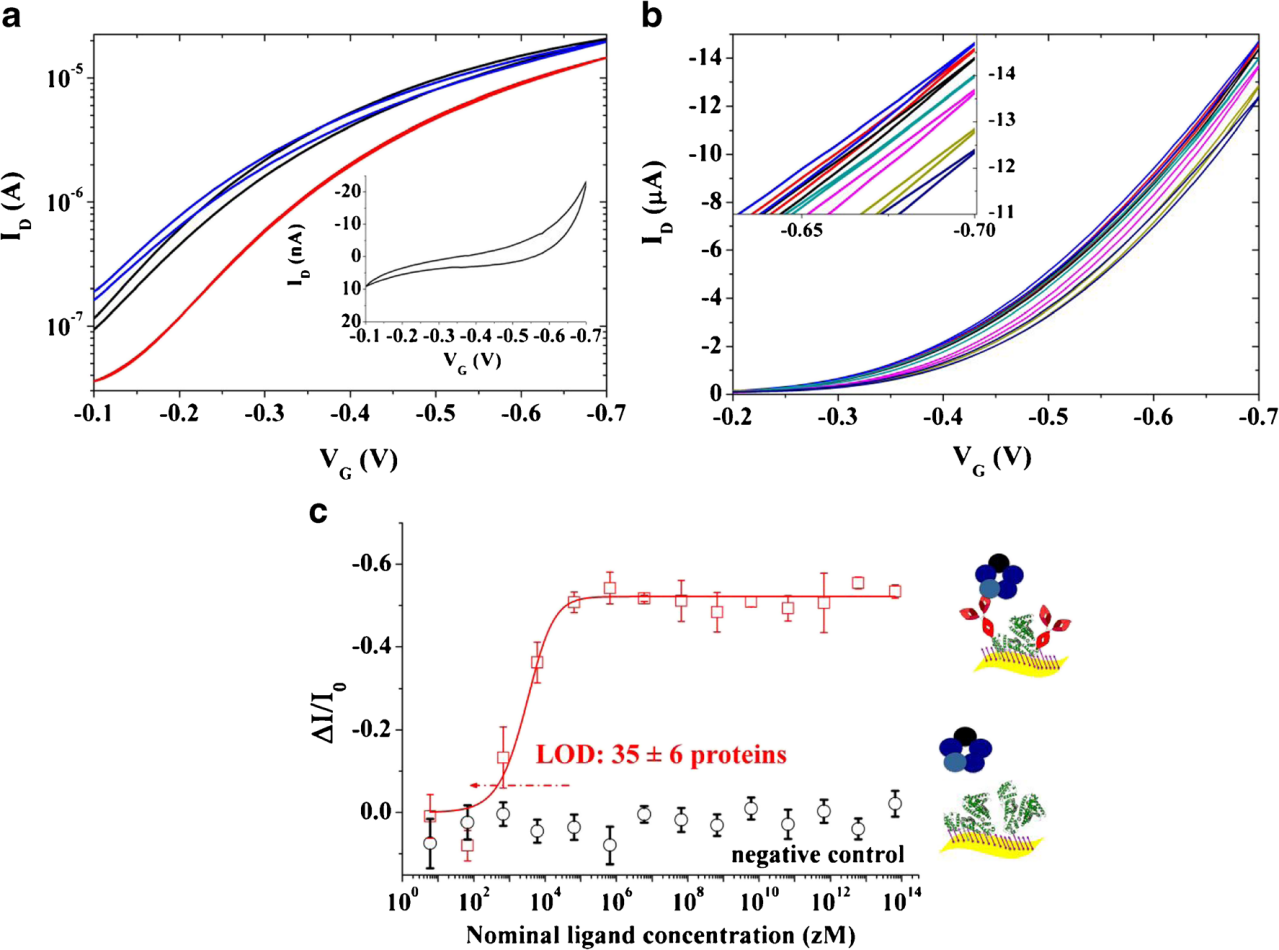
**a** Transfer I–V curves (*I*_D_ vs. *V*_G_ ranging from − 0.1 to − 0.7 V at *V*_D_ = − 0.4 V) comprising a bare Au gate (black and blue curves) or the SAM one (red curve). The black and blue curves are taken on the same SiMoT device before and after measuring the whole sensing calibration. The *I*_G_ for the SAM gate is shown as the inset. **b** SiMoT sensing transfer characteristics (*I*_D_ vs. *V*_G_ at *V*_D_ = − 0.4 V). The red curve corresponds to the anti-CRP functionalized gate incubated in the sole PBS solution. The same gate is further exposed, in sequence, to PBS standard solutions of CRP at concentrations of 6 zM (black curve), 60 zM (blue curve), 6 · 10^2^ zM (dark cyan curve), 6 · 10^3^ zM (magenta curve), and 6 · 10^6^ zM (dark-green curve). In the inset, a zoom into the high *V*_G_ range is reported. **c** The CRP/anti-CRP dose curve (red squares) measured in PBS is shown as the relative change of the *I*_D_ current (Δ*I*/*I*_0_) vs. the CRP concentration. A functionalized gate comprising both the anti-CRP and the BSA is used. The black circles are the negative control responses of a bare BSA-functionalized gate to CRP in PBS standard solutions. The full red line is the fitting of the CRP binding curve. The data points are relevant to three replicates of the dose curves, while the error bars have been evaluated as the relative standard deviation

Figure 1d shows the *I*_D_ − *V*_D_ output characteristics of the SiMoT device measured sweeping *V*_D_ from 0 to − 0.5 V and with *V*_G_ ranging from 0 to − 0.5 V. A good field effect current modulation has been achieved with a negligible level of hysteresis, as demonstrated by the back sweep current. The SiMoT response towards the CRP in PBS has been reliably evaluated by measuring the device transfer characteristics (*I*_D_ vs. *V*_G_ sweeping from − 0.1 to − 0.7 V, at *V*_D_ = − 0.4 V) before and after exposure to target analyte. All the data discussed from here on were gathered on SiMoT device appropriately cycled to reach the transistor operational stability [36]. In fact, the organic semiconductor requires a stabilization step before reaching a stable operation. This goal has been achieved by repeated cycling of the transfer curves until an overlap of at least three subsequent traces was obtained. In particular, the black curve in Fig. 2a is relevant to the transfer curve measured on a bare gold gate after the aforementioned stabilization of the organic semiconductor. The current registered when the gate is functionalized with the SAM is shown in red. A shift of the threshold voltage (*V*_T_) of 100 mV has been measured. The blue curve is relevant to the transfer curve measured on the same device with the very same gold gate used to measure the black curve, but after acquiring the whole CRP dose curve. As it is evident, the blue and black curves almost overlap. In fact, the transfer curves of the bare gold gate measured at the beginning and at the end of the sensing showed a relative current change of less than − 5%. This control experiment allows to assess that only the sensing experiments involving a negligible *I*_D_ current decreases of the bare gold gate, presumably ascribable to a minor degradation of the transport properties of the organic semiconductor, are suitable. Such a procedure rules out that the current changes measured during the biosensing experiments could be attributed to SiMoT device performance degradation. The curve in the inset of Fig. 2a is relevant to the gate current *I*_G_, which is three orders of magnitude lower than *I*_D_. It is worth mentioning that all the curves have been measured in forward and reverse mode to evidence the occurrence of hysteresis, minimized by tuning the voltage ranges in order to prevent any electrochemical process. The CRP detection is performed by measuring the SiMoT transfer characteristics after incubation of the anti-CRP functionalized gate electrode in 100 μl of PBS standard solutions of CRP ranging from 6 zM (zeptomolar, 10^−21^) to 6 10^14^ zM (600 pM) nominal concentrations. The PBS solution reproduces a physiologically relevant environment, holding a pH of 7.4 and an ionic-strength of 162 mM. Figure 2b shows typical sensing transfer characteristics, measured after incubation of the same anti-CRP functionalized gate electrode into progressively more concentrated CRP standard solutions. In the inset of Fig. 2b, the sensing transfer characteristics at high *V*_G_ are zoomed in. The red curve, taken as the *baseline*, is measured after incubation of the functionalized gate in bare PBS solution. The black and blue curves, measured subsequently, correspond to the incubation in the 6 zM and 60 zM CRP solution, respectively. The red, black, and blue curves do not show any significant difference. The dark cyan curve has been registered after incubation in a CRP nominal concentration of 6 10^2^ zM. It is now evident that a significant current decreases with respect to the baseline, reproduced as the standard solutions at increased CRP concentration are progressively assayed, until the saturation of the response is achieved. The SiMoT responses (relative current variations) to CRP in PBS standard solutions are shown in Fig. 2c as red squares, while the black circles are the responses to CRP assayed with a bare BSA-functionalized gate electrode. The response has been evaluated from the measured transfer characteristics according to the following equation:

5 $$ \frac{\Delta I}{I_0}=\frac{I-{I}_0}{I_0} $$

where *I* is the drain current measured after incubation with the target CRP concentration and *I*_0_ is the *baseline* drain current, both taken at *V*_G_ that maximizes the transconductance *g*_m_ *=* δ*I*_D_/δ*V*_G_. The relative standard deviation of the individual calibration points, measured on three devices fabricated on different chips measured with three different gates, ranged between 1 and 6%, indicating a good interdevice reproducibility. Moreover, the negative control experiment in Fig. 2c (black circles) strikingly shows no significant response to CRP assayed with a gold gate electrode functionalized with the sole BSA. In fact, the high selectivity of the SiMoT biosensing platform is effectively proven by the almost zero response of the control assay in the whole concentration range. The limit of detection (LOD) has been computed, being the concentration that leads to a response of (Δ*I*/*I*_0_)_mean_*± kσ*, where (Δ*I*/*I*_0_)_mean_ is the average response of the blank sample, *σ* is the relative standard deviation, and *k* is a numerical factor chosen according to the level of confidence required. IUPAC recommends a value of *k* = 3 as the probability of a blank signal being 3-fold higher than the (Δ*I*/*I*_0_)_mean_ (i.e., a false positive) is less than 1% [2]. According to this definition, a LOD level of 9.8% has been computed from the noise level of the control experiment. The estimated LOD level corresponds to a CRP nominal concentration of 590 zM. The nominal number of CRP proteins (#CRP) is given by:

6 $$ \#\mathrm{CRP}=c{N}_{\mathrm{A}}V $$

where *c* is the nominal ligand concentration expressed in molarity, *N*_A_ is the Avogadro number, and *V* is the volume of solution in which the gate is incubated, in our case 100 μl. Therefore, a CRP nominal concentration of 590 zM corresponds to 35 ± 6 proteins. The full red line shown in Fig. 2c has been obtained with an ad hoc studied SiMoT dose curve modeling that has been discussed in details elsewhere [30, 37]. The proposed model is based on the Poisson distribution probability to describe the occurrence of few biding events. In fact, it is assumed that the SAM on the gate is composed by domains of highly densely packed capturing antibodies, electrostatically connected through an H-bond network. This network is present both in the chem-SAM and in the bio-SAM. If one CRP protein binds to any of the capturing antibodies populating one domain, the entire domain changes its work-function due to a collective cooperation mechanism enabled by the hydrogen bonding network. It has been assumed that the process is irreversible. Therefore, if other CRP bindings occur within the same domain, no other change in work-function is possible. The propagation of the work-function change is limited by defect. Thus, a compact, uniform, and electrostatically connected SAM is necessary for the propagation. The more compact and defect-free the SAM is, the larger the domain generated upon interaction with one ligand is and the higher the sensing response will be. This mechanism has been assumed to be at the basis of the amplification process, along with the field effect amplified response, allowing the SiMoT single-molecule detection.

**Fig. 3:**
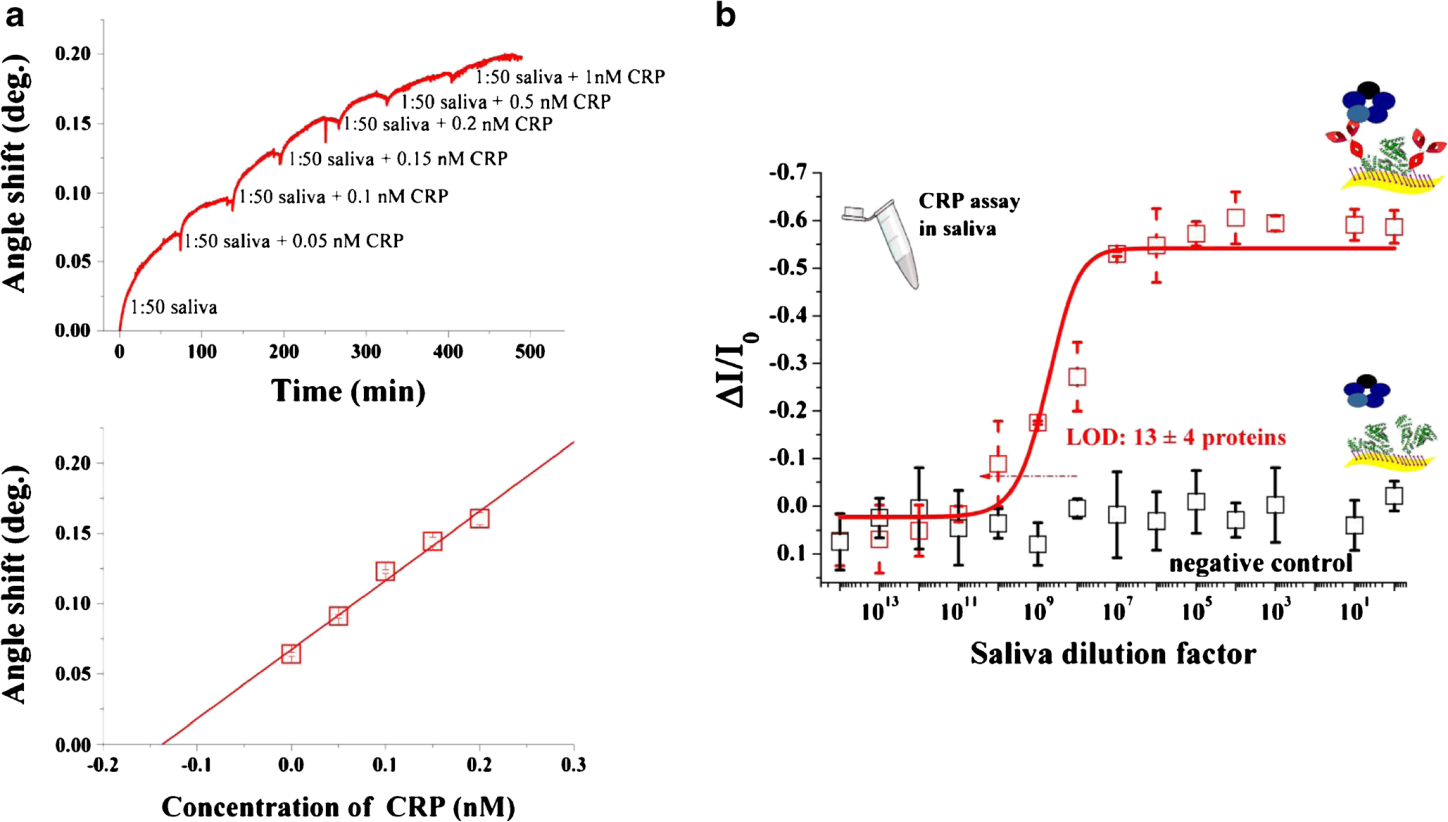
**a** Binding of various concentrations of CRP in diluted human saliva to 0.1 mg/ml anti-CRP immobilized onto Au slide along with the calibration curves for various concentrations of CRP. **b** CRP detection in a diluted sample of human saliva. The CRP/anti-CRP dose curve (red squares) measured in diluted human saliva is shown as the relative change of the *I*_D_ current (Δ*I*/*I*_0_) vs. the saliva dilution factor. A functionalized gate comprising both the anti-CRP and the BSA is used. The black circles are the negative control responses of a bare BSA-functionalized gate to CRP in diluted human saliva. The full red line is the result of the SiMoT modeling. The data points are relevant to three replicates of the dose curves, while the error bars have been evaluated as the relative standard deviation

CRP has been assayed in diluted saliva, voluntarily donated by one of the authors. The human saliva was diluted with PBS to obtain a stock solution of saliva in a ratio of 1:50. To minimize matrix effects, present in saliva, the standard addition method was used. Hence, stock solutions containing the same amount of diluted saliva were spiked with known amounts of CRP in order to yield spiked saliva samples with analyte concentrations in the range of 0.05–1 nM. The amount of CRP in human saliva was measured by means of SPR measurements performed by loading aliquots (100 μl) of CRP-spiked human saliva to a gold-coated glass SPR slide previously functionalized with the anti-CRP. The antibody-coated gold slides were incubated with the analyte solution at 20 °C for at least 1 h before rinsing with aliquots of PBS to remove any unbounded protein. The resulting sensograms were obtained by recording the reflection angle as a function of time at 670 nm. All measurements have been made in triplicate using different SPR SAM-coated slides. Figure 3a shows a representative sensogram of the binding of different concentrations of CRP on the surface of the SAM-coated slide along with the calibration curves obtained using the measured change of reflection angle. The range of concentration shown is limited to the range where the response is linear with the concentration of the analyte. The calibration graphs were used for direct determination of CRP in human saliva yielding a concentration of 7 ± 1 nM. The standard deviation is calculated according to Eq. 7 [38].

7 $$ {s}_{xE}=\frac{S_{y/x}}{b}\sqrt{\frac{1}{n}+\frac{{\overline{y}}^2}{b^2{\sum}_i{\left({x}_i-\overline{x\ }\right)}^2}} $$

where *s*_*xE*_ is the standard deviation of the extrapolated *x* value *x*_*E*_, *s*_*y/x*_ is the random error in the y direction, *b* is the slope of the line, $\overline{y}$ and $\overline{x}$ are the mean values of *y* and *x*, and *n* is the total number of measurements. The 95% confidence interval corresponds to *x*_*E*_ ± *t*_(*n*−2)_*s*_*xE*_ = 7 ± 2 nM.

The resultant number of CRP proteins, evaluated applying Eq. 6, is equal to (4 ± 2) 10^11^ molecules in 100 μl of human saliva. Therefore, the whole saliva has been diluted at 1:10^15^ in PBS, to guarantee the absence of endogenous CRP at the single-molecule level. The CRP dose–response in human saliva has been measured incubating the anti-CRP functionalized gate into progressively less-diluted saliva samples with exactly the same protocol used for CRP detection in PBS standard solutions. The resulting dose–response is reported in Fig. 3b as red squares. Each data point is averaged over three replicates, measured with three different gates on three different devices. The red full line has been obtained with the SiMoT dose curve model carried out with the same set of parameters computed for the calibration dose curve in PBS. It is apparent an excellent level of agreement between the dose curves measured in PBS and in diluted human saliva. It is worth mentioning that the calibration curves obtained with the SPR experiments and with the SiMoT device are relevant to two different analyte concentration range, thus holding different binding isotherms. In particular, while Langmuir isotherm holds for the systems described by the law of mass action [3] and can be used to describe the SPR ligand–receptor binding reaction, it is not suitable to describe the SiMoT calibration curves. In fact, in this latter case, the ligand–receptor binding reaction is definitely far from equilibrium and an ad hoc analytical function that models the calibration curves measured with the SiMoT has been developed [31, 37]. The model is based on the Poisson distribution probability to better account few binding events. It also provides a model for the amplification mechanism occurring in the SAM, responsible for the single-molecule detection and it is described in details elsewhere [31, 37]. The negative control experiment has been performed using a bare BSA-functionalized gate. The selectivity of the SiMoT device has been proven even in real sample, as no significant response has been registered. Such a negative control experiment has been designed considering that a catalog of 2290 proteins found in whole saliva has been reported [11]. Therefore, BSA is expected to be more effective than anti-IgM or anti-IgG capturing SAMs to minimize non-specific binding in human saliva. In fact, it has been already proven elsewhere that endogenous immunoglobulin G (IgG) in diluted human saliva binds to anti-human-IgG bio-SAM deposited on the gate of a the SiMoT [31]. Consequently, a BSA-functionalized gate has been herein exploited to assess non-specific binding of endogenous interferences, such as human-IgGs or human-IgMs, that should be present at high concentrations in human saliva. The LOD level associated with the dose curve measured in diluted saliva, evaluated considering the corresponding negative control experiment level of noise, is 6.2%. The LOD level corresponds to a dilution of 1:(3.07 ± 0.75) 10^10^. This datum is in excellent agreement with the total number of CRP molecules originally present in the saliva sample and strikingly proves that single-molecule quantitative detection is possible also in saliva, although a dilution is required in order to reduce the endogenous CRPs to the physical limit. In fact, the SiMoT configuration allowed label-free selective CRP detection in human saliva with a LOD as low as 13 ± 4 proteins. Moreover, the results obtained for the detection of CRP in human saliva samples demonstrate that the developed SiMoT biosensing platform is suitable for the analysis in real samples. The developed SiMoT biosensor is label-free, compatible with low-cost fabrication procedures and allows the reliable and ultra-highly sensitive detection of CRP at the physical limit in real samples, such as human saliva. Besides, the SiMoT fabrication procedure is fully compatible with printing technologies employed for electronic device fabrication. This achievement represents an extraordinary breakthrough that will set the ground for a revolution in early point-of-care clinical diagnostics.

## Conclusions

A label-free electronic biosensing platform based on the SiMoT technology for CRP detection at physical limit has been herein successfully demonstrated in human saliva. Besides, the SiMoT device presented is not only highly sensitive but also highly selective. This work represents a first proof-of-principle of single-molecule detection in human saliva. Indeed, it paves the way to fundamental clinical applications, where the onset of pathological states requires the detection of clinically relevant biomarkers in complex samples at the physical limit. This is the case of the majority of protein biomarkers of cancer, neurological disorders or viral infections [39]. Furthermore, the SiMoT biosensing platform format is compatible with the development of label-free electronic assays where multiple target analytes can be measured simultaneously in clinical relevant fluids, such as saliva, but also tears and urine.
